# Microbial and Metabolic Gut Profiling across Seven Malignancies Identifies Fecal *Faecalibacillus intestinalis* and Formic Acid as Commonly Altered in Cancer Patients

**DOI:** 10.3390/ijms25158026

**Published:** 2024-07-23

**Authors:** Maria Kulecka, Paweł Czarnowski, Aneta Bałabas, Maryla Turkot, Kamila Kruczkowska-Tarantowicz, Natalia Żeber-Lubecka, Michalina Dąbrowska, Ewa Paszkiewicz-Kozik, Jan Walewski, Iwona Ługowska, Hanna Koseła-Paterczyk, Piotr Rutkowski, Anna Kluska, Magdalena Piątkowska, Agnieszka Jagiełło-Gruszfeld, Michał Tenderenda, Cieszymierz Gawiński, Lucjan Wyrwicz, Magdalena Borucka, Maciej Krzakowski, Leszek Zając, Michał Kamiński, Michał Mikula, Jerzy Ostrowski

**Affiliations:** 1 Department of Gastroenterology, Hepatology and Clinical Oncology, Centre of Postgraduate Medical Education, 02-781 Warsaw, Poland; 2 Department of Genetics, Maria Sklodowska-Curie National Research Institute of Oncology, 02-781 Warsaw, Poland; 3 Department of Cancer Prevention, Maria Sklodowska-Curie National Research Institute of Oncology, 02-781 Warsaw, Poland; 4 Department of Internal Medicine and Hematology, Military Institute of Medicine—National Research Institute, 04-141 Warsaw, Poland; 5 Department of Lymphoid Malignancies, Maria Sklodowska-Curie National Research Institute of Oncology, 02-781 Warsaw, Poland; 6 Early Phase Clinical Trials Unit, Maria Sklodowska-Curie National Research Institute of Oncology, 02-781 Warsaw, Poland; 7 Department of Soft Tissue/Bone Sarcoma and Melanoma, Maria Sklodowska-Curie National Research Institute of Oncology, 02-781 Warsaw, Poland; 8 Department of Breast Cancer & Reconstructive Surgery, Maria Sklodowska-Curie National Research Institute of Oncology, 02-781 Warsaw, Poland; 9 Department of Oncological Surgery and Neuroendocrine Tumors, Maria Sklodowska-Curie National Research Institute of Oncology, 02-781 Warsaw, Poland; 10 Department of Oncology and Radiotherapy, Maria Sklodowska-Curie National Cancer Research Institute, 02-781 Warsaw, Poland; 11 Department of Lung and Chest Cancer, Maria Skłodowska-Curie National Research Institute of Oncology, 02-781 Warsaw, Poland; 12 Department of Gastrointestinal Surgical Oncology, Maria Sklodowska-Curie National Research Institute of Oncology, 02-781 Warsaw, Poland

**Keywords:** *Faecalibacillus intestinalis*, cancer patients, shotgun metagenomics, metabolomics

## Abstract

The key association between gut dysbiosis and cancer is already known. Here, we used whole-genome shotgun sequencing (WGS) and gas chromatography/mass spectrometry (GC/MS) to conduct metagenomic and metabolomic analyses to identify common and distinct taxonomic configurations among 40, 45, 71, 34, 50, 60, and 40 patients with colorectal cancer, stomach cancer, breast cancer, lung cancer, melanoma, lymphoid neoplasms and acute myeloid leukemia (AML), respectively, and compared the data with those from sex- and age-matched healthy controls (HC). α-diversity differed only between the lymphoid neoplasm and AML groups and their respective HC, while β-diversity differed between all groups and their HC. Of 203 unique species, 179 and 24 were under- and over-represented, respectively, in the case groups compared with HC. Of these, *Faecalibacillus intestinalis* was under-represented in each of the seven groups studied, *Anaerostipes hadrus* was under-represented in all but the stomach cancer group, and 22 species were under-represented in the remaining five case groups. There was a marked reduction in the gut microbiome cancer index in all case groups except the AML group. Of the short-chain fatty acids and amino acids tested, the relative concentration of formic acid was significantly higher in each of the case groups than in HC, and the abundance of seven species of *Faecalibacterium* correlated negatively with most amino acids and formic acid, and positively with the levels of acetic, propanoic, and butanoic acid. We found more differences than similarities between the studied malignancy groups, with large variations in diversity, taxonomic/metabolomic profiles, and functional assignments. While the results obtained may demonstrate trends rather than objective differences that correlate with different types of malignancy, the newly developed gut microbiota cancer index did distinguish most of the cancer cases from HC. We believe that these data are a promising step forward in the search for new diagnostic and predictive tests to assess intestinal dysbiosis among cancer patients.

## 1. Introduction

The gut microbiota harvests nutrients and energy from the diet, trains the immune system, protects against opportunistic pathogens, and produces metabolites with local and systemic actions [1]. Microbial profiles defined by richness, diversity, and composition are modulated by several variables, including host genotype, age and sex, lifestyle, diet, physical activity, sanitation, and many others. In addition, the gut microbiota is considered a potential environmental factor associated with different human pathologies, including cancer [1,2,3,4,5,6,7], acting through endogenous metabolites and microbial products such as short-chain fatty acids (SCFAs), amino acids, secondary bile acids, and lipopolysaccharides.

An imbalance in gastrointestinal microbial community complex, known as dysbiosis, has been linked to various disorders, including obesity, diabetes, cardiovascular disorders, cancer, hypertension and inflammatory bowel disease (IBD) [8,9,10,11,12]. For instance, IBDs like Crohn’s disease and ulcerative colitis are linked to reduced diversity of beneficial bacteria, such as *Faecalibacterium prausnitzii*, and an increase in harmful species like *Escherichia coli* [13]. Obesity and metabolic disorders also exhibit microbial imbalances, typically showing a higher ratio of Firmicutes to Bacteroidetes [14]. Specific bacteria, such as *Akkermansia muciniphila*, which is associated with a healthy gut lining and improved metabolic health, are often found in lower abundance in obese patients [15]. Conversely, increased levels of *Prevotella* and *Ruminococcus* have been observed, which may contribute to increased energy harvest from the diet [16]. In conditions like irritable bowel syndrome (IBS), a decrease in *Lactobacillus* and *Bifidobacterium* species is common, while overgrowth of methane-producing bacteria like *Methanobrevibacter smithii* is linked to constipation [17]. Additionally, dysbiosis is connected to mental health disorders, including depression and anxiety, through the gut–brain axis. Reduced levels of *Bifidobacterium* and *Lactobacillus* species, which produce neuroactive compounds, correlate with increased symptoms of these disorders [18].

In cancer patients, the intestinal microbiota modulates the host metabolic, inflammatory and immune responses to microbial-derived metabolites and carcinogens, all of which may enhance or diminish disease development and progression [19]. A clear example is the association between alterations of the gut microbiota community and the onset of colorectal cancer (CRC); an increased abundance of *Bacteroides*, *Parvimonas*, *Bilophila*, and *Fusobacterium*, and a decreased abundance of *Ruminococcus*, *Bifidobacterium*, and *Streptococcus* species in those with gastrointestinal (GI) malignancies have been identified as factors that modulate local immune responses and production of bacterial genotoxins [1,20,21,22,23,24,25,26,27]; however, gut dysbiosis also plays a critical role in development or prevention of many other neoplasms, including breast and lung cancers, melanoma, lymphoma, and leukemia [28,29,30,31]. Some changes in the gut microbiome may be common to different types of neoplasms.

In this study, we used whole-genome shotgun sequencing (WGS) and gas chromatography/mass spectrometry (GC/MS) to define common changes in the composition of the gut microbiota, and identified distinct fecal metabolomic profiles (i.e., SCFAs and amino acids) in seven different types of human malignancy.

## 2. Results

### 2.1. Patients Overview

This study investigated 340 patients (207 women and 133 men) who were diagnosed with CRC, stomach, breast, and lung cancer, melanoma, lymphoid neoplasms, or AML, and 178 (91 women and 87 men) HC. Considering sex- and age-related differences in the intestinal microbiota [32], HCs for each subgroup were age- and sex-matched (Table 1). Pretreatment fecal samples were collected from all patients before systemic treatment.

### 2.2. Metagenomic and Metabolomic Analyses of Pretreatment Fecal Samples

DNA isolated from fecal samples was analyzed using WGS-based metagenomic sequencing. On average, 14 million reads were generated per sample (median, 13 million). Five (Bacteroidota, Bacillota, Actinomycetota, Pseudomonadota, Verrucomicrobiota) out of the 67 identified phyla had an abundance of >1% within the microbiome. Our datasets identified a total of 260 species present in more than 0.01% of reads. Top ten abundant species are from the following genera: *Bacteroides*/*Phocaeicola* (*B. uniformis*, *P. vulgatus*, *P. dorei* and *B. stercoris*) and *Alistipes* (*A. onderdonkii* and *A. shahii*). The four remaining top species are *Escherichia coli*, *Prevotella copri*, *Faecalibacterium prausnitzii* and *Akkermansia muciniphila*. They are present in 47% of all the reads.

#### 2.2.1. Bacterial Diversity

**Figure 1:**
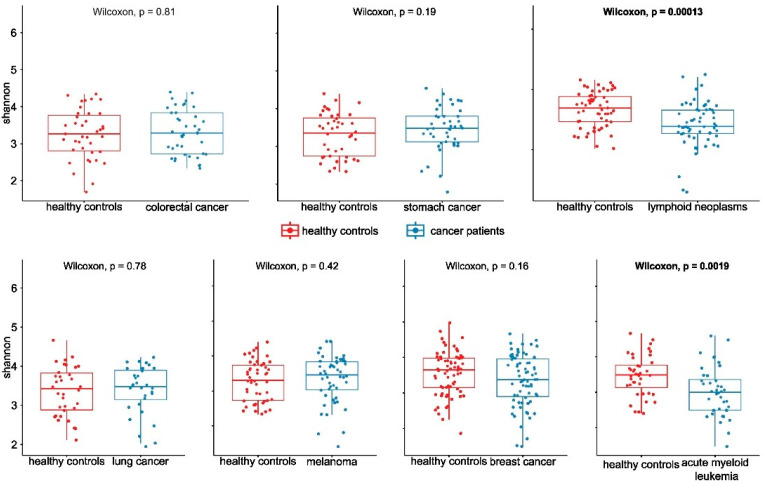
α-diversity of the gut microbiome population of patients versus their corresponding healthy controls.

**Figure 2:**
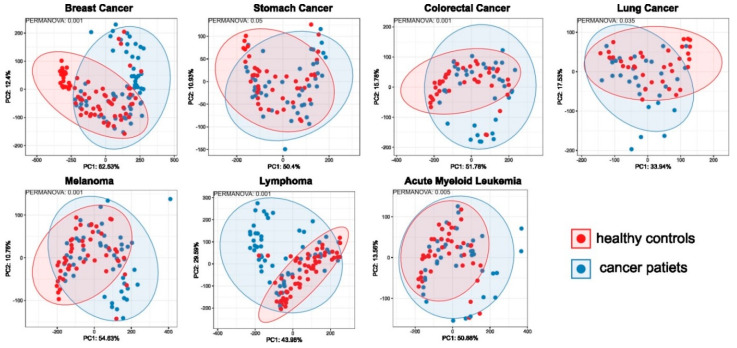
β-diversity, as measured by principal component analysis (PCA), revealed significant differences between each of the patient groups and their corresponding controls.

The structure of the bacterial community among pretreatment fecal samples was evaluated by analyzing the α- and β-diversity at the species level. The α-diversity was analyzed using the Shannon index, a marker of bacterial richness and evenness. The β-diversity was analyzed using principal component analysis (PCA). As shown in Figure 1, after multiple hypothesis testing corrections, the estimated Shannon index for each comparison revealed lower α-diversity of the gut microbiota only in lymphoid neoplasm and AML samples compared with their corresponding HC. In turn, PCA indicated that the stool microbiome of the seven groups was significantly different from that of their corresponding HCs (Figure 2).

#### 2.2.2. Taxonomic Profiling

**Figure 3:**
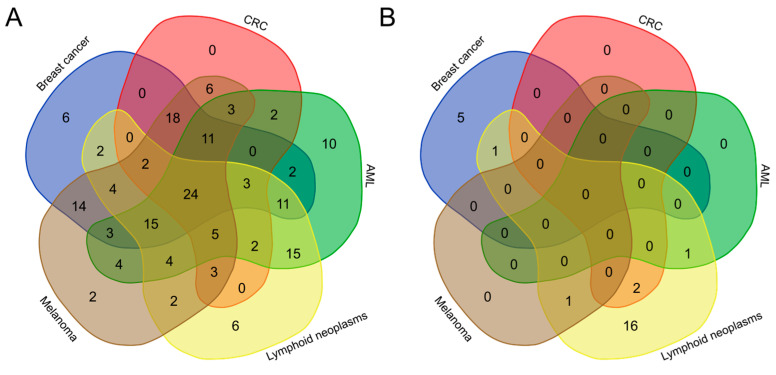
Venn diagram showing (**A**) under-abundant and (**B**) over-abundant species identified by the seven pairwise comparisons. The number in each cell indicates the number of unique taxa shared among neoplasm types.

To reduce the uncertainty of taxonomic classification due to low read counts during differential taxonomic analyses, the Mann–Whitney U-test was performed in two separate analyses based on species with a relative abundance of >50 reads in the case or corresponding control samples. We identified 203 unique species that showed a significant difference in abundance in at least one of the groups (adjusted *p*-value < 0.05), of which 179 and 24 species were under- and over-represented, respectively, when compared with the HC. Pairwise comparisons between the breast cancer, CRC, AML, lymphoid neoplasm, and melanoma groups and their corresponding HCs revealed a reduced abundance of 115, 79, 114, 98, and 120 species (Figure 3A), and increased abundance of 6, 2, 1, 18, and 1 species, respectively (Figure 3B). In patients with lung or stomach cancer, only two species were less abundant, and none were more abundant than in their corresponding HC; however, an additional 31 species tended to be under-represented in patients with stomach cancer (adjusted *p*-value < 0.1) (Tables S1 and S2).

*Faecalibacillus intestinalis* was under-represented in each of the seven groups studied, whereas Anaerostipes hadrus was under-represented in all but the stomach cancer group, and 22 species (*Anaerobutyricum hallii*, *Blautia pseudococcoides*, *Blautia hansenii*, *Blautia* sp. *SC05B48*, *Blautia wexlerae*, *Blautia obeum*, *Butyrivibrio crossotus*, *Clostridioides difficile*, *Coprobacter fastidiosus*, *Coprococcus eutactus*, *Coprococcus catus*, *Coprococcus* sp. *ART55/1*, *Dorea formicigenerans*, *Dorea longicatena*, *Eubacterium ventriosum*, *Faecalibacterium* sp. *IP-3-29*, *Faecalibacterium duncaniae*, *Faecalibacterium* sp. *HTF-F*, *Faecalitalea cylindroides*, *Lachnospira eligens*, *Qiania dongpingensis*, *Roseburia* sp. *NSJ-69*) were under-represented in patients with CRC, breast cancer, melanoma, AML and lymphoid neoplasms. Of these, 23 belonged to the phylum Firmicutes, and one to the phylum Bacteroidetes.

In turn, of 24 species that were uniquely differentially more abundant and enriched in cases than in HC, 18 (*Citrobacter portucalensis*, *Shigella boydii*, *Shigella flexneri*, *Shigella sonnei*, *Citrobacter braakii*, *Shigella dysenteriae*, *Klebsiella variicola*, *Klebsiella michiganensis*, *Klebsiella oxytoca*, *Enterobacter hormaechei*, *Klebsiella quasipneumoniae*, *Escherichia* sp. *E4742*, *Escherichia marmotae*, *Klebsiella aerogeneswere*, *Enterobacter cloacae*, *Escherichia fergusonii*, *Escherichia albertii*, *Citrobacter freundii*), 6 (*Streptomyces lydicus*; *Eggerthella guodeyinii*, *Pseudomonas aeruginosa*, *Arabiibacter massiliensis*, *Bifidobacterium pseudolongum*, *Enterobacter cloacae*), 2 (*Escherichia fergusonii*, *Escherichia albertii*), 1 (*Klebsiella pneumoniae*), and 1 (*Citrobacter freundii*) were enriched only in patients with lymphoid neoplasms, breast cancer, CRC, melanoma, or AML, respectively. Twenty species belonged to the phylum Proteobacteria (most to the *Enterobacteriaceae* family and the three genera *Escherichia*, *Enterobacter*, and *Klebsiella*), and four belonged to the diverse phylum Actinobacteria.

To sum up, although 179 species showing significant differences in abundance were associated with normal samples compared with samples from patients with breast cancer, CRC, AML, lymphoid neoplasm, and melanoma, few were exclusive to each pairwise comparison. Instead, of 24 species associated with case samples, 14, 5, and 1 were exclusive to lymphoid neoplasms, breast cancer and melanoma, respectively, while only 4 were common to lymphoid neoplasms, 1 to breast cancer, 2 to CRC, and 1 to AML (Figure 3).

**Figure 4:**
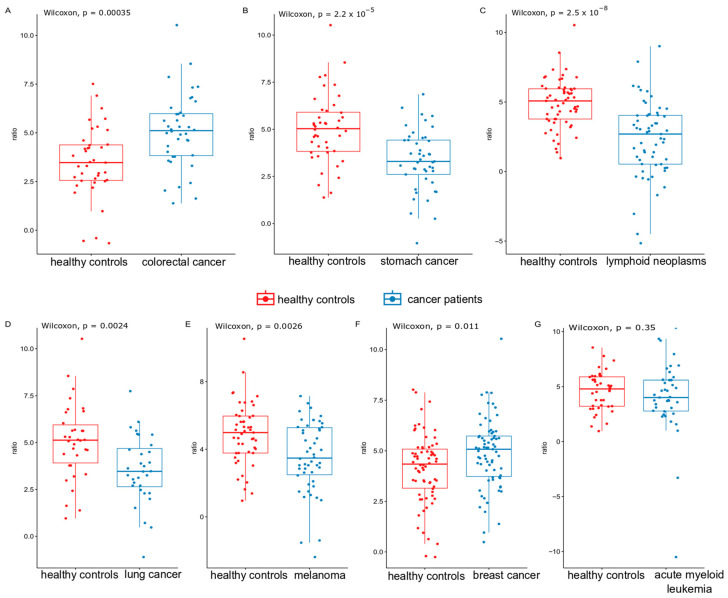
The microbiome cancer index was used to compare the microbiome population in the gut of patient groups and their corresponding healthy controls. Panels from A to G represent comparisons between healthy controls and subsequent cancer types: (**A**)—colorectal cancer, (**B**)—stomach cancer, (**C**)—lymphoid neoplasms, (**D**)—lung cancer, (**E**)—melanoma, (**F**)—breast cancer, (**G**)—acute myeloid leukemia.

Next, based on the ratio of bacterial species over-represented in HCs to those over-represented in the neoplastic gut ecosystems estimated for each sample, we created a “gut microbiome cancer index”. In contrast to the Shannon index, the estimated gut microbiome index for each comparison was significantly lower (in all but the AML group) than that for their corresponding HCs (Figure 4).

#### 2.2.3. Correlation between Bacteria Populations and Metabolites

A sufficient number of fecal samples was available from all cases, but only from 45 of the HC; therefore, contrary to the metagenomic study, we were unable to select appropriate control subgroups for metabolomic analyses that could be matched to the age and sex of each subgroup of patients. Therefore, we used the whole HCs group as the reference group. Metabolites isolated from fecal samples were analyzed using mass GC spectrometry, which revealed the profiles of seven SCFAs (acetic acid, butanoic acid, formic acid, hexanoic acid, isobutyric acid, pentanoic acid, propanoic acid) and nine amino acids (AAs) (alanine (Ala), glycine (Gly), glutamic acid (Glu), isoleucine (Ile), leucine (Leu), methionine (Met), phenylalanine (Phe), proline (Pro) and valine (Val)).

**Figure 5:**
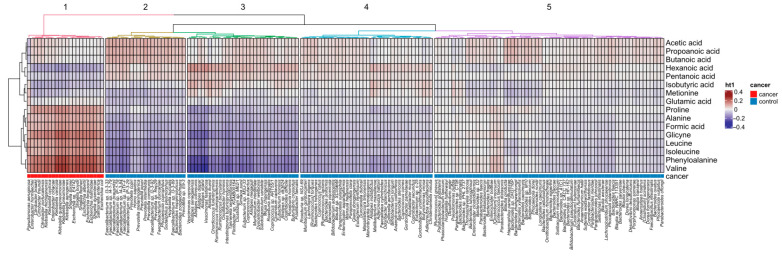
Heat map derived from pairwise correlations (Spearman’s coefficient) between the abundance of bacterial species and metabolites identified in healthy controls and case-mixed cancer patients.

First, we calculated all pairwise correlations between the abundance of bacterial species and each metabolite in fecal samples from HCs and case-mixed cancer patients by calculating Spearman’s coefficient; the magnitude of individual values within a dataset was visualized in a heatmap. The distribution of correlations formed five separate bacterial species clusters and two metabolite clusters, the first of which comprised six SCFAs (acetic acid, propanoic acid, butanoic acid, pentanoic acid, hexanoic acid, and isobutyric acid) and two AAs (Glu and Met), and the second of which comprised the remaining AAs and formic acid (Figure 5). Although the strength of most of the relationships between taxa and metabolites was non-existent or weak, that between *Klebsiella variicola*, *Klebsiella quasipneumoniae*, *Klebsiella aerogenes*, *Klebsiella pneumonie*, *Shigella sonnei*, *Shigella boydii*, *Sigella flexaneri*, *Shigella dysenteriae*, *Escherichia alberti*, *Escherichia fergusonii*, and *Escherichia marmotae* from cluster 1, which are the predominated taxa over-represented in case-mixed cancer patients, showed a strong positive correlation with fecal Val, Phe, Gly and Pro levels, and a negative correlation with hexanoic acid levels. Of the bacteria over-represented in control samples, *Alistipes senegalensis*, *Alistipes communis*, *Alistiper dispar*, *Alistipes shahii*, *Vescimonas coprocola*, and *Vescimonas fastidiosa* showed a strong negative correlation with Val, Phe, Gly and Pro levels, and a positive correlation with hexanoic acid levels, while seven *Faecalibacterium species* (cluster 2) showed a strong negative correlation with most AAs and a positive correlation with acetic, propanoic, and butanoic acid levels.

#### 2.2.4. Fecal SCFA and Amino Acid Profiling

**Figure 6:**
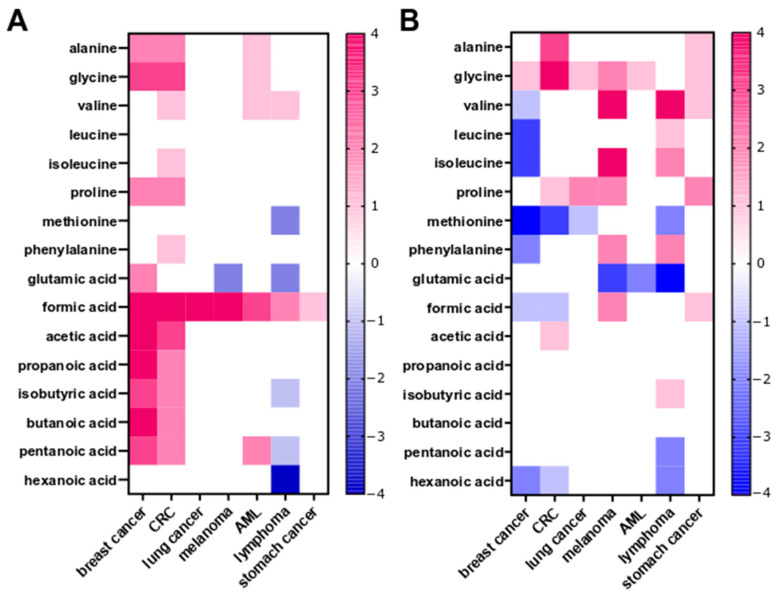
The heat map showing changes in SCFA and amino acid concentrations (**A**) and in the percentage amounts of these metabolites (**B**) across fecal samples from seven groups of patients compared with normal fecal samples; 1—*p* < 0.05; 2—*p* < 0.01; 3—*p* < 0.001; 4—*p* < 0.0001.

Next, we used two methods to analyze metabolomic profiles; first we compared the relative concentrations of metabolites per gram of stool weight (Figure 6A), and second, we compared the contribution of a given metabolite to the overall profile of a sample, calculated as a percentage of the total SCFA and amino acid concentration in the stool sample tested (Figure 6B).

In the first comparison, the formic acid concentration was significantly higher (adjusted *p*-value < 0.05) in fecal samples from each of the seven groups of patients with a malignancy than in samples from the HC. An increase in the concentrations of five other SCFAs (acetic, propanoic, isobutyric, butanoic and pentanoic acids) and three AAs (Ala, Gly, and Pro) was detected in fecal samples from patients with breast cancer and CRC. In patients with lymphoid neoplasms, the concentrations of fecal isobutyric, pentanoic, and hexanoic acids, and of Met and Glu, were higher than in HC. In the other groups, especially patients with lung cancer, stomach cancer and melanoma, the concentrations of most fecal metabolites were no different from those in the HCs (Figure 6A).

The analysis of metabolite proportions revealed that the patterns between stool samples from the case groups and HCs were different from those observed after analysis of metabolite concentrations. Differences were related primarily to AAs, and most were observed in patients with breast cancer, melanoma, or AML, and to a lesser degree in patients with CRC and stomach cancer. The type of malignancy affected the proportions of Gly, Val, Ile, Pro, Met, and Glu that were observed mostly in breast cancer, melanoma and lymphoid neoplasm patients, whereas only the proportion of hexanoic acid was different in patients with breast cancer, colorectal, or lymphoid neoplasms (Figure 6B).

### 2.3. Metagenomic and Metabolomic Analyses to Compare Pretreatment and Post-Treatment Fecal Samples

While the gut microbiota in pretreatment samples was highly variable among patients with different neoplasm, neither chemotherapy nor immunotherapy altered the bacterial α-diversity, as assessed by the Shannon index, and there was only a minor difference in the β-diversity between pre- and post-treatment samples. In addition, taxonomic analyses did not identify any bacteria that differentiated melanoma or lung cancer patients tested before and after immunotherapy, or patients with hematological malignancies tested before and after chemotherapy. Only in breast cancer patients after chemotherapy was there a tendency towards differences in the abundance of five bacteria (*Blautia* sp. *SC05B48*, *Anaerostipes rhamnosivorans*, *Campylobacter jejuni*, *Nocardioides* sp. *BP30*, *Roseburia hominis*) (padj. between 0.062 and 0.077). In addition, the abundance of only one bacterium, *Actinomyces oris*, differed (padj. = 0.027) between pretreatment samples from those collected 12–24 months after the end of treatment.

### 2.4. Functional Analyses

The MetaCyc Metabolic Pathway Database, which allows reconstruction of metabolic networks from sequenced genomes, was used to identify 160 MetaCyc pathways that met the criteria for statistical analysis. Of these, there were 1, 5, and 8 differentiated patients with breast cancer, AML, or lung cancer, while 23, 44, 79, and 154 differentiated patients with melanoma, CRC, stomach cancer, or lymphoma, respectively, from the HCs (Table S3).

**Figure 7:**
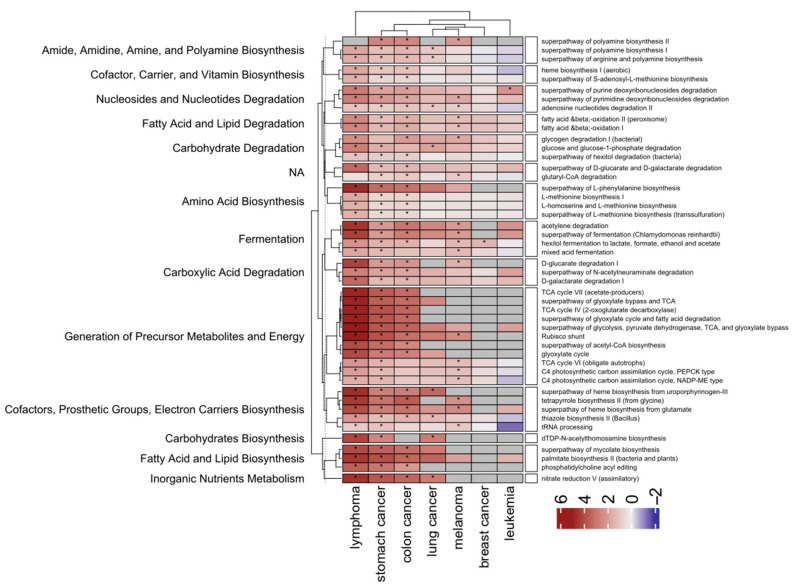
The MetaCyc Metabolic Pathways that differentiate each of the case groups from their corresponding control, grouped according to their superpathways. The heatmap presents log2-transformed data. Red and blue denote pathways that are over- and under-represented, respectively, in a case group compared with the control group; gray denotes lack of occurrence of a given pathway in a case group. * denotes statistical significance (Mann–Whitney U-test).

There were 2 pathways (P461-PWY: hexitol fermentation to lactate, formate, ethanol and acetate and SALVADEHYPOX-PWY: adenosine nucleotides degradation II) and 15 pathways (ARG+POLYAMINE-SYN: superpathway of arginine and polyamine biosynthesis; FAO-PWY: fatty acid &beta;-oxidation I; GLUCARDEG-PWY: D-gluconate degradation I; P161-PWY: acetylene degradation; POLYAMSYN-PWY: superpathway of polyamine biosynthesis I; PWY-5136: fatty acid &beta;-oxidation II (peroxisome); PWY-5189: tetrapyrrole biosynthesis II (from glycine); PWY-5675: nitrate reduction V (assimilatory); PWY-5723: Rubisco shunt; PWY-5918: superpathway of heme biosynthesis from glutamate; PWY-6891: thiazole biosynthesis II (Bacillus); PWY0-1297: superpathway of purine deoxyribonucleosides degradation; PWY0-1298: superpathway of pyrimidine deoxyribonucleosides degradation; PWY0-1415: superpathway of heme biosynthesis from uroporphyrinogen-III; and PWY4LZ-257: superpathway of fermentation (*Chlamydomonas reinhardtii*)) that were identified in five and four case groups, respectively, and 30, 41 and 72 pathways were identified in three, two and one group, respectively (Table S3). Of these, the most abundant differential pathways (i.e., found at least in three case groups) are involved in generation of precursor metabolites and energy; cofactors, prosthetic groups, electron carriers’ biosynthesis; fatty acid and lipid biosynthesis; amide, amidine, amine, and polyamine biosynthesis; fermentation; carboxylic acid degradation; carbohydrate degradation; and nucleoside and nucleotide degradation. All were over-represented in the patient groups (Figure 7).

## 3. Discussion

Analysis of high-throughput sequencing data using a bioinformatics pipeline is the method of choice when looking for differences in microbial richness, diversity, and composition [33]. By implementing a sequence curation pipeline optimized for analysis of WGS-based datasets, we focused on identifying common and distinct taxonomic configurations among two GI and five extra-GI malignancies [34]. Since the microbiota modulates sex- and age-related changes in innate immunity, inflammation, and cognitive function [19,26], datasets from patients with each type of neoplasm were compared with respective healthy individuals who were matched by age and sex.

A decrease in α-diversity was identified only between patients with lymphoid neoplasms or AML and their corresponding HC, whereas we found differences in β-diversity between all malignancy groups studied and their corresponding HC. Taxonomic profiling identified changes in the relative abundance of taxa between cases and HC, albeit to varying degrees; of 203 unique species, between 2 and 179 showed significant differences in abundance among the studied groups. Of these, *Faecalibacillus intestinalis* was under-represented in each of the seven groups studied, *Anaerostipes hadrus* was under-represented in all but the stomach cancer group, and 22 species were under-represented in breast cancer, CRC, melanoma, AML, and lymphoid neoplasms. *Faecalibacillus intestinalis* [35], *Blautia* genus [36], *Coprobacter* genus [37], and *Faecalibacterium* sp. [38] are obligate anaerobic genera present in the normal human gut flora. *Anaerostipes hadrus*, *Anaerobutyricum hallii*, and *Coprococcus catus* are SCFA-producing bacteria [39,40,41]. The relationship between *Butyrivibrio crossotus*, *Ruminococcus* sp., and *Dialister* may play a role in the balance between T helper cell type 1 (Th1) and Th2 inflammatory responses [42]. Both *Faecalibacterium duncaniae* (formerly known as *F. prausnitzii*) and *Eubacterium ventriosum* are considered to be colorectal-protecting microorganisms with anti-inflammatory properties [43,44]. Thus, most species showing reduced abundance in our case groups can be considered to promote a healthy status.

By contrast, species such as *Enterobacter cloacae* complex, *Enterobacter hormaechei* [45,46,47], *Escherichia albertii* [48], *Klebsiella michiganensis* [49], *K. variicola* [50], *Pseudomonas aeruginosa* [51], *Shigella dysenteriae*, *S. flexneri*, *S. boydii* and *S. sonnei* [52], *Citrobacter braakii*, *C. freundii* and *C. portucalensis* [53,54], *Klebsiella pneumoniae*, *K. variicola*, and *K. quasipneumoniae* [55,56], showing a higher abundance in fecal samples from case groups than in samples from HCs are considered to be significant pathogenic factors responsible for severe and often opportunistic GI, urinary, pulmonary, and blood infections. These disease-associated species are often commensal, but may become pathogenic in a disease-associated environment or they may be pathogens that occur naturally in low abundance in a healthy microbiome [1].

Metagenome functional content of the different taxonomic profiles was assessed using the MetaCyc Metabolic Pathway Database [57]. Of 160 MetaCyc pathways identified, there were 1, 5, 8, 23, 44, 79, and 154 differentiated patients with breast cancer, AML, lung cancer, melanoma, CRC, stomach cancer, or and lymphoma, respectively, from their corresponding HC. There were 2, 15, 30, 41, and 72 pathways identified in five, four, three, two, and one case groups, respectively. Of the most abundant differential pathways found at least in the three case groups, all were over-represented in patient groups and were related to energy metabolism, nucleotide degradation, fatty acid and lipid degradation, and fermentation.

Recently, the functional consequences of changes in the microbial community were annotated to 20 MetaCycle modules that showed differential abundance between Chinese patients with locally advanced rectal cancer responding or not responding to chemoradiotherapy [58]. Modules included mixed acid fermentation and guanosine diphosphate-mannose biosynthesis, which could retard tumor growth and increase cell death in response to chemotherapy by impairing glucose metabolism via the tricarboxylic acid (TCA) cycle, glycolysis, and the pentose phosphate pathways [59]. Increased nucleotide metabolism can support the uncontrolled growth of tumor cells by generating pyrimidine and purine bases for DNA replication and cellular bioenergetics [60,61]. Two hexitol metabolism-related pathways, the superpathway of hexitol degradation (HEXITOLDEGSUPER-PWY) and the hexitol fermentation to lactate, formate, ethanol and acetate pathway (P461-PWY), were associated with decreased risk of gastric cancer [62], and their increased abundance a marker of immune activation in patients with chronic granulomatous disorders [63]. The polyamine biosynthesis II superpathway (POLYAMINSYN3-PWY) is also associated with decreased gastric cancer risk, and two types of (TCA) cycle (Krebs cycles II and VII) were associated with the risk of gastric cancer [62].

Lipid biomolecules such as phospholipids, fatty acids, triglycerides, sphingolipids, cholesterol, and cholesteryl esters serve as building blocks for the plasma membrane and various cellular structures, and play roles as secondary messengers [64]; they are also a source of energy, and all may be linked to the onset of tumors [65]. Sterols and isoprenoids produced through the mevalonate pathway contribute to formation and progression of tumors [65]. Cancer cell survival and metastasis also depend on the uptake and utilization of exogenous fatty acids (FAs), mainly through FA β-oxidation (FAO) pathways [66], deregulation of which has been confirmed in various human malignancies [67]. While the oxidation of long-chain FA can be inhibited by butyrate generated by the gut microbiota [68], FAO undergoes reprogramming in immune cells, as well as other cancer-associated host cells that potentially create a tumor-supportive environment [69]. Whereas alterations in FAO may be related to inflammatory bowel disease and development of colon tumors [70], we found that the “fatty acid&beta; oxidation I and II” pathways were significantly over-represented in patients with CRC, stomach cancer, lymphoma neoplasms, or melanoma.

The exact microbial species and microbiota-dependent mechanisms that affect cancer development and progression are not fully understood [71]. Although bacterial species such as *Fusobacterium nucleatum*, *Escherichia coli*, *Bacteroides fragilis*, *Aspergillus*, *Clostridium septicum*, *Enterococcus faecalis*, and *Streptococcus bovis* are known to drive colorectal carcinogenesis [20,21,24], most species associated with CRC are observed in only a minority of datasets [1,2]. A recently published study [1] that integrated seven public datasets containing WGS sequencing data derived from fecal samples obtained from CRC patients and normal individuals identified 11 species and 54 species that were under-represented and over-represented, respectively, in cancer samples compared with normal samples. By contrast, our study identified 79 species that were under-represented and only two that were over-represented mixed-case samples. Only two species, *Faecalibacillus intestinalis* and *Anaerostipes hadrus*, were under-represented in our cases and those evaluated by Riveros Escalona et al. [1]. Furthermore, although changes in the metagenomic profiles that accompany cancer treatment are considered obvious, and have been confirmed by many previous studies [72], we did not confirm these commonly reported findings. Unfortunately, we do not have a reason for this surprising discrepancy between our data and those of others.

Differences in the abundance of bacterial groups can alter their functional redundancy, which in turn can change the metabolic function of the gut microbiota [73]. SCFAs and branched chain FA, alcohols, ammonia, amines, sulfur compounds, phenols and indoles, glycerol, and choline derivatives, all of which exert local and systemic effects, are degradation products of dietary carbohydrates, lipids and proteins generated by the intestinal microbiota [74]. Of these, SCFAs are the most abundant, serving as energy sources, acting to improve the integrity of the intestinal barrier, and exerting anti-inflammatory effects [75,76], whereas bacterial metabolic processes in distal parts of the colon may be related to the availability of AAs [77]. The abundance of most of the species over-represented in our case samples correlated positively with fecal Val, Phe and Gly levels, and negatively with hexanoic acid levels; the opposite correlations were found for bacterial species that were over-represented in control samples. A subcluster of seven species of *Faecalibacterium* correlated negatively with most AAs and with formic acid, and positively with the levels of acetic, propanoic, and butanoic acid.

Formic acid concentrations were significantly higher in fecal samples from the seven case groups than in their corresponding controls. Formate is an intermediate metabolite of one-carbon metabolism, and a mediator of metabolic interactions between mammalian organisms, diet, and the gut microbiome [78]. Being a by-product of anaerobic fermentation by some species of intestinal bacteria, formic acid enters the circulation to boost the endogenous formate pool. Bacterial oxidation of formate and aerobic respiration, accompanied by increased levels of formic acid in the gut lumen, may be signatures of inflammation-associated gut dysbiosis [79,80].

In a previous study we demonstrated that the relative levels of seven out of nine assayed fecal SCFAs differentiated at least two groups of diarrheal patients from HCs [81]. Formic acid and caproic acid were more abundant, and pentanoic acid was less abundant, in each of the three diarrhea groups (i.e., case-mix cancer, inflammatory bowel disease, and *Clostridioides difficile*-infected patients). Five AAs differentiated at least two patient groups from HC. Of these, the levels of glycine and valine were highest, and those of methionine and glutamic acid were lowest [81]. In the current study, we found that fecal formic acid levels were significantly higher in each of the seven case groups, and increased levels of acetic, propanoic, isobutyric, butanoic, and pentanoic acids, as well as Ala, Gly, and Pro, were found in patients with breast cancer and CRC. In the lymphoid neoplasm group, increased levels of isobutyric, pentanoic, and hexanoic acids, as well as Met and Glu, were documented; however, the concentrations of most fecal metabolites in patients with lung cancer, stomach cancer, or melanoma did not differ from those in controls.

The human gut microbiota comprises at least 1800 genera and 15,000–36,000 bacterial species in low or high abundance [82,83]; all of these bacteria co-evolve with the host, although only a fraction of these will be present in a single individual [84]. Thus, the composition of the microbiome is characterized by enormous inter- and intra-individual complexity and variability; however, to ensure the functional stability and resilience of the microbiome, different bacterial groups (at the species and strain level) are responsible for the same biological processes [1,85,86,87,88]. In turn, loss of disease-associated functional redundancy is characterized by differences in taxonomic abundance [89]. There are two main methods for studying microbial communities: marker gene analyses, which are based on the sequencing of a gene-specific region of genomes (e.g., hypervariable regions of the 16S bacterial rRNA gene), and WGS [90,91,92]. Of these, marker gene sequencing can detect only a fraction of the gut microbiota community, whereas untargeted WGS can identify less abundant taxa and allows assignment of taxonomy at both the species and strain levels [93]. Although both approaches have been used extensively to characterize tumor-associated microbial communities [92], the results are highly variable. The question remains: how do we objectively distinguish a healthy microbiome from an unhealthy one?

Newly emerging methods that analyze the relationship between bacterial taxonomic and functional profiles are trying to address these challenges [94]. The Gut Microbiome Health Index (GMHI) [95], which was formulated using 50 microbial species selected from 4347 human stool metagenomes that represent healthy and unhealthy conditions, distinguished healthy from unhealthy groups regardless of clinical diagnosis, with a precision of 73.7%. The improved version of the GDHI, the Gut Microbiome Wellness Index (GMWI2) [29], is based solely on gut taxonomic signatures. The other index, which expands on the GMHI, is the hiPCA [91], which monitors the framework of personalized health status by analyzing the contribution of species in different groups of patients. Other methodologies such as the Lasso penalized logistic regression model [29] or Random Forest-based machine learning classifiers [19] have also been employed. These microbiome-related health indices are based on species richness and depend on taxonomic classification. Recently, an index based on functional characteristics rather than on the taxonomic composition of the gut microbiome was proposed [31]. Our own gut microbiome cancer index was created by calculating the ratio between the number of microbial species over-represented in control samples and the number of species that were over-represented in neoplastic gut ecosystems. Compared with that in the corresponding HC, the index was significantly lower in all groups of patients, except for the AML group.

Without a doubt, metagenomic results are highly dependent on the sequencing technology and bioinformatic pipeline used [96]. Assessment of the composition and diversity of 16S sequencing data, which used four different bioinformatic pipelines (mothur, QIIME, kraken, and CLARK), revealed that targeted metagenomics offers the opportunity to demonstrate that trends in changes in bacterial profiles, rather than accurate and objective differences, correlate with disease [33]. Similar conclusions can be drawn from comparison of the gut microbiota of breast cancer patients using WGS datasets based on selected marker genes [33], and whole sequences of the bacterial genome (this study). The results of the two analyses are not comparable.

In summary, development of targeted metagenomics approaches requires advances in both large-scale sequencing technology and processing of sequencing data [97]; each data processing step can introduce bias, thereby affecting the biological interpretation of the sequencing results [33]. In this study, we used a sequence curation pipeline optimized for analyses of WGS-based datasets to identify taxonomic and metabolomic profiles among seven groups of malignancies. With the exception of *Faecalibacillus intestinalis*, which was under-represented, and formic acid, whose relative concentration was significantly higher in all case groups than in HC, we found more differences than similarities between the studied groups, with great variability in diversity, taxonomic/metabolomic profiles, and functional assignments.

Since sequencing was carried out virtually at the same time and in the same reference laboratory, and bioinformatic analyses of the obtained sequences were carried out using the same analytical pipeline, it can be assumed that introduction of bias was minimized [33]; however, the variable number of patients in each study group may have affected the power of statistical testing, and the results obtained may demonstrate trends rather than objective differences that correlate with different types of malignancy. Nevertheless, the newly developed gut microbiota cancer index was able to distinguish all groups of cases, except AML, from HC. We believe that this type of analysis represents a step in the right direction with respect to the search for new diagnostic and predictive tests to assess the role of intestinal dysbiosis in disease.

## 4. Materials and Methods

### 4.1. Patients

This study was conducted in accordance with the ethical standards of the institutional and/or national research committees, and in accordance with the 1964 Helsinki Declaration and its later amendments (or comparable ethical standards) and was approved by Maria Sklodowska-Curie National Research Institute of Oncology Local Bioethics Board (decision 40/2018). All participants provided informed consent to participate.

Between July 2018 and December 2022, 340 mix-case neoplasm patients were recruited (Table 1). The majority were newly diagnosed at any disease stage, and some (mostly lymphoma and melanoma patients) were in relapse within 1 to 3 years from the last treatment. Clinical information was obtained from the institutional medical record management system. Sex- and age-matched healthy controls (HCs), assigned separately to each of the studied patient groups (Table 1), were either hospital staff or were recruited during cancer screening programs who declared a good health condition and remained on a diet without specific restrictions. None of the participants and controls had used antibiotics within 2 months before pretreatment fecal sampling or had inflammatory bowel disease or a history of cancer.

Fecal samples were self-collected using a stool specimen collection kit, as described previously [98]; 340 pretreatment samples were obtained before systemic oncological treatment, and 165 post-treatment samples were obtained after completing the final or one cycle of chemotherapy or immunotherapy. For 41 breast cancer patients, post-treatment samples were collected after completing neoadjuvant and/or adjuvant therapy, which included the TCH-regimen (docetaxel, carboplatin, trastuzumab), the TCH-P regimen (docetaxel, carboplatin, trastuzumab, pertuzumab), or the ACdd regimen (doxorubicin, cyclophosphamide), and samples from another 12 patients were collected 12–24 months after completion of the last treatment cycle. For 25 patients with acute myeloid leukemia (AML), post-treatment samples were obtained after completing standard induction or consolidation treatment (cytarabine + idarubicin, cytarabine + daunorubicin, or cytarabine + gemtuzumab ozogamicin) either with or without autologous hematopoietic stem cell transplantation (autoHCT). The case-mixed lymphoid neoplasm group comprised 28 large B cell lymphomas, 12 Hodgkin lymphomas, 13 multiple myelomas, and 7 other lymphoma types. Post-treatment samples were collected from 21 patients after standard chemotherapy specific to their disease stage and lymphoma subtype, and from 18 patients after completing high-dose chemotherapy before autoHCT. In total, 27 and 21 fecal samples were collected following immunotherapy for melanoma (nivolumab or nivolumab + ipilimumab) or lung cancer (nivolumab), respectively. Clinical information was obtained from the institutional medical record management system.

### 4.2. Metagenomics Analysis

DNA was isolated from fecal samples using the QIAamp Fast DNA Stool Mini Kit protocol (Qiagen, Hilden, Germany) and quantified using fluorimetry with the Qubit dsDNA High Sensitivity Assay (Thermo Fisher Scientific, Carlsbad, CA, USA). Metagenomic sequencing was conducted on the Illumina NovaSeq 6000 platform (San Diego, CA, USA) using 10 ng of isolated DNA. The sequencing protocol involved 100-base pair paired-end reads, and standard procedures recommended by the manufacturer were followed [99].

### 4.3. SCFA and Amino Acid Profiling

Metabolites were extracted from frozen stool samples, derivatized, and subjected to gas chromatographic analysis on an Agilent 7000D Triple Quadrupole mass spectrometer coupled to a 7890 GC System with a G4513A autosampler (Agilent Technologies, Santa Clara, CA, USA), as described [81,98].

### 4.4. Statistical Analysis

#### 4.4.1. Bacteria and Metabolites

Shannon diversity indices were calculated by the iNEXT package version 3.0. Values were compared using the Kruskal–Wallis test or Mann–Whitney U-test (two groups only). Bacterial taxa were assigned using Kraken2 version 2.1.3, with default parameters and databases. Species-level assignments were made by Bracken version 2.7 using minimum number of counts of 100. Differences in taxa abundance between groups were assessed using the LINDA [Linear (Lin) Model for Differential Abundance (DA)] [100] method for compositional data, with *p*-values corrected using the Benjamini–Hochberg [101] procedure to minimize the false discovery rate (FDR). Differences in metabolite concentrations between study groups were assessed using the Mann–Whitney U-test.

#### 4.4.2. Associations between Bacteria and Metabolites

Taxa non-ambiguously associated with at least one metabolite were identified by the metadeconfoundR package. Only taxa with more than 1000 assigned reads (on average) and present in at least 10% of samples were analyzed. Regularised Canonical Correlation Analysis was performed on these taxa and their metabolites using the Ridge method, with parameters tuned as described in the mixOmics tutorial [102]. The correlation structure was visualized by the complexHeatmap package. Bacterial species were clustered using Ward’s method (“ward.D2” method in base R hclust function). The optimal number of modules was selected using the dynamicTreeCut package.

Functional assignment was conducted by HUMAnN version 3.0 (part of BioBakery Workflows) [103], using MetaCyc [57] pathways as a reference database. Quality filtering and decontamination were performed by KneadData as a part of the functional analysis. The LINDA method was used to assess compositional data, with *p*-values corrected by the Benjamini–Hochberg procedure to minimize the FDR.

## Figures and Tables

**Table 1:** The enrolled cases and sex-and age-matched healthy controls.

Groups	Cases		Controls	
	Women	Men	Women	Men
	*n*/Median; Range (Years)	*n*/Median; Range (Years)	*n*/Median; Range (Years)	*n*/Median; Range (Years)
Colorectal cancer	20/66; 36–82	20/67; 35–82	20/68; 49–79	20/61; 50–81
Stomach cancer	15/68; 37–78	30/68; 40–87	15/70; 37–82	30/62; 41–81
Breast cancer	71/50; 30–79		71/54; 30–82	
Lung cancer	17/64; 54–81	17/61; 35–85	17/64; 52–82	17/61; 40–81
Melanoma	23/65; 48–84	27/66; 34–88	23/65; 47–82	27/60; 42–81
Lymphoid neoplasms	35/58; 22–78	25/57; 31–74	35/59; 22–82	25/58; 30–81
Acute myeloid leukemia	26/60; 20–68	14/51; 23–74	26/60; 22–73	14/50; 23–75

## Data Availability

The datasets presented in this study can be found in online repositories. The names of the repository/repositories and accession number(s) can be found below: https://www.ncbi.nlm.nih.gov/ (accessed on 17 July 2024), PRJNA1116523 (https://dataview.ncbi.nlm.nih.gov/object/PRJNA1116523?reviewer=b48031qdle9932pdanc95dk6ok, accessed on 17 July 2024). Healthy controls data is available at PRJNA885289. Breast cancer patient data are available at PRJNA1001944.

## Supplementary Materials

The following supporting information can be downloaded at: https://www.mdpi.com/article/10.3390/ijms25158026/s1.
